# Identification of INOSITOL PHOSPHORYLCERAMIDE SYNTHASE 2 (IPCS2) as a new rate‐limiting component in Arabidopsis pathogen entry control

**DOI:** 10.1111/tpj.70159

**Published:** 2025-04-29

**Authors:** Josephine Mittendorf, Tegan M. Haslam, Cornelia Herrfurth, Nicolas Esnay, Yohann Boutté, Ivo Feussner, Volker Lipka

**Affiliations:** ^1^ Department of Plant Cell Biology, Albrecht‐von‐Haller‐Institute for Plant Sciences Georg‐August‐University Goettingen Goettingen D‐37077 Germany; ^2^ Department of Plant Biochemistry, Albrecht‐von‐Haller‐Institute for Plant Sciences University of Goettingen Goettingen D‐37077 Germany; ^3^ Service Unit for Metabolomics and Lipidomics, Goettingen Center for Molecular Biosciences (GZMB) University of Goettingen Goettingen D‐37077 Germany; ^4^ Laboratoire de Biogenèse Membranaire Université de Bordeaux, UMR5200 CNRS Villenave d'Ornon France; ^5^ Department of Plant Biochemistry, Goettingen Center for Molecular Biosciences (GZMB) University of Goettingen Goettingen D‐37077 Germany; ^6^ Central Microscopy Facility of the Faculty of Biology & Psychology Georg‐August‐University Goettingen Goettingen 37077 Germany

**Keywords:** disease resistance, sphingolipids, endomembrane trafficking, pathogen entry control, powdery mildew

## Abstract

INOSITOL PHOSPHORYLCERAMIDE SYNTHASE 2 (IPCS2) is involved in the biosynthesis of complex sphingolipids at the *trans*‐Golgi network (TGN). Here, we demonstrate a role of IPCS2 in penetration resistance against non‐adapted powdery mildew fungi. A novel *ipcs2*_W205_* mutant was recovered from a forward genetic screen for Arabidopsis plants with enhanced epidermal cell entry success of the non‐adapted barley fungus *Blumeria graminis* f. sp. *hordei* (*Bgh*). A yeast complementation assay and a sphingolipidomic approach revealed that the *ipcs2*_W205_* mutant represents a knock‐out and lacks IPCS2‐specific enzymatic activity. Further mutant analyses suggested that IPCS2‐derived glycosyl inositol phosphorylceramides (GIPCs) are required for cell entry control of non‐adapted fungal intruders. Confocal laser scanning microscopy (CLSM) studies indicated that upon pathogen attack, IPCS2 remains at the TGN to produce GIPCs, while focal accumulation of the defense cargo PENETRATION 3 (PEN3) at *Bgh* penetration sites was reduced in the *ipcs2*_W205_* mutant background. Thus, we propose a model in which sorting events at the TGN are facilitated by complex sphingolipids, regulating polar secretion of PEN3 to host‐pathogen contact sites to terminate fungal ingress.

## Significance Statement

Polarized transport of executive defense gene products to sites of attempted microbial invasion is important for plant pathogen entry control and disease resistance. Here, we provide evidence that INOSITOL PHOSPHORYLCERAMIDE SYNTHASE 2 (IPCS2)‐dependent sphingolipid production contributes to the role of the *trans*‐Golgi network (TGN) as a multi‐domain sorting compartment, and mediates proper delivery of an ATP‐binding cassette transporter to polarized plasma membrane domains at plant–microbe interaction sites.

## INTRODUCTION

Plants have developed an efficient defense machinery to terminate the attack of non‐adapted pathogens. As a first obstacle, pathogens encounter preformed physical and chemical barriers such as waxy cuticles, rigid cell walls, and antimicrobial compounds (Nürnberger & Lipka, 2005; Thordal‐Christensen, 2003). If intruders are able to successfully overcome these defense barriers, host cell surface‐located pattern recognition receptors (PRRs) recognize conserved pathogen‐associated molecular patterns (PAMPs) or host‐derived danger‐associated molecular patterns (DAMPs), triggering active defense responses (Boller & Felix, 2009; Jones & Dangl, 2006).

As obligate biotrophic pathogens, powdery mildew fungi derive nutrients from living host cells and cause disease on a wide range of plants, including economically important crops. During the asexual life cycle, conidiospores germinate on the plant leaf surface and produce appressoria to penetrate the epidermal cell wall. Upon fungal entry attempts, the host plant dynamically rearranges the cytoskeleton, aggregates organelles, and secretes antifungal compounds to form defense structures called papillae underneath host‐pathogen contact sites (Hückelhoven & Panstruga, 2011; Lipka et al., 2008). Apoplastic papillae consist of callose, reactive oxygen species (ROS), phenolics, and defense molecules, providing physical and chemical barriers for penetration resistance against fungal intruders (Ellis, 2006; Meyer et al., 2009; Thordal‐Christensen et al., 1997; Underwood & Somerville, 2008). However, if the fungal pathogen is able to successfully breach the plant cell wall, haustoria are formed that are required for host nutrient uptake and effector secretion (Micali et al., 2008). As a host cell response, post‐penetration mechanisms are activated, which typically include the generation of callose‐enriched haustorial encasements and programmed cell death (PCD) of invaded cells (Lipka et al., 2005; Meyer et al., 2009).

PENETRATION 1 (PEN1), PEN2, PEN3, and PEN4 were identified in forward genetic screens as molecular components of Arabidopsis cell entry control against non‐adapted powdery mildew fungi (Collins et al., 2003; Hématy et al., 2020; Lipka et al., 2005; Stein et al., 2006). PEN1 is a plasma membrane (PM)‐resident SOLUBLE N‐ETHYLMALEIMIDE‐SENSITIVE‐FACTOR ATTACHMENT RECEPTOR (SNARE) protein, which is required for vesicle fusion events to ensure papilla formation (Assaad et al., 2004; Collins et al., 2003). A second, PEN1‐independent pathway deals with the polarized secretion of antifungal compounds to sites of pathogen ingress. The mitochondria‐associated atypical myrosinase PEN2 and the phytochelatin synthase PEN4 were reported to catalyze the generation of potentially toxic indole glucosinolate‐derived molecules (Bednarek et al., 2009; Fuchs et al., 2016; Hématy et al., 2020). These antifungal products are believed to be secreted by the ATP binding cassette (ABC) transporter protein PEN3 across the PM to the apoplastic battlefield to terminate fungal penetration attempts (Bednarek et al., 2009; Hématy et al., 2020; Stein et al., 2006).

Interestingly, PM‐recycled and not *de novo* synthesized PEN1 and PEN3 were found to be recruited to sites of attempted fungal invasion and strongly accumulate within apoplastic papillae (Meyer et al., 2009; Reichardt et al., 2011; Underwood & Somerville, 2013). Further studies regarding the involvement of actin filaments revealed that polarized secretion of PEN1 and PEN3 follows distinct trafficking pathways (Qin et al., 2021; Underwood & Somerville, 2013). However, the *trans*‐Golgi‐network (TGN)‐based proteins aminophospholipid ATPase 3 (ALA3) and ECHIDNA (ECH) were reported to be required for delivery of both PEN1 and PEN3 to the cell surface (Liu et al., 2023; Uemura et al., 2019; Underwood et al., 2017). Thus, a model was suggested in which the receiving of recycled PEN1 and PEN3 is facilitated by a common TGN compartment, and from there splits into separate pathways for the polarized secretion of both defense proteins to sites of attempted fungal invasion (Nielsen, 2024). In alignment with this hypothesis, the GDSL‐lipase/esterase family protein GOLGI DEFECTS 36 (GOLD36), which alters the lipid composition at the TGN, was found to be a specialized component in the delivery of PEN3 to apoplastic papillae (Underwood, 2022).

Upon infection with a non‐adapted powdery mildew, the nanodomain‐localized REMORIN 1.3 (REM1.3) protein accumulates in papillary membranes, providing evidence that these include some types of nanodomains (Xing et al., 2019). Further, PEN1 has been reported to co‐localize with REM1.3 in papillae nanodomains (Xing et al., 2019). As nanodomains are important for membrane subcompartmentalization to act as, *inter alia*, signaling platforms in response to different stimuli (Jaillais et al., 2024; Liang et al., 2018; Ma et al., 2022; Platre et al., 2019; Smokvarska et al., 2020), they could here serve as transfer stations delivering immunity cargo such as PEN1 and PEN3 to extracellular papillae. Some plant membrane nanodomains are lipid‐ordered and rely on sterol‐ and sphingolipid composition (Gronnier et al., 2017; Grosjean et al., 2015; Lv et al., 2017). Although PEN3 was never shown to be part of nanodomains via co‐localization studies using nanodomains marker proteins, biochemical analysis suggests that the membrane environment of PEN3 might be sterol‐ and sphingolipid‐enriched (Kierszniowska et al., 2009; Minami et al., 2009). The most abundant sphingolipids at the PM are glycosyl inositol phosphorylceramides (GIPCs) (Bahammou et al., 2024). GIPCs contain a ceramide backbone, which consists of a saturated or monounsaturated very‐long‐chain fatty acid (VLCFA) and a poly‐hydroxylated long‐chain base (LCB) moiety, modified with a polar sugar headgroup (Haslam & Feussner, 2022; Luttgeharm et al., 2016; Mamode Cassim et al., 2020). The first committed step in GIPC production is catalyzed by inositol phosphorylceramide synthases (IPCS1‐3), which transfer a head group from phosphatidylinositol (PI) onto ceramide to produce the intermediate inositol phosphorylceramide (IPC) at the TGN (Ito et al., 2021; Mina et al., 2010), which is then further modified by glycosylation events. Biophysical analyses revealed that GIPCs influence membrane properties by increasing the thickness and electronegativity of the PM outer leaflet (Mamode Cassim et al., 2021). Moreover, previous studies provided evidence that IPCS2‐derived GIPCs facilitate polar secretion of the auxin efflux carrier PIN2 to apical membranes, suggesting that GIPCs are involved in secretory trafficking (Ito et al., 2021; Markham et al., 2011; Wattelet‐Boyer et al., 2016).

Here, we demonstrate a direct link between sphingolipid metabolism and Arabidopsis cell entry control. Genetic interference of *IPCS2* results in a defective penetration resistance against non‐adapted powdery mildews. Mutant analyses combined with sphingolipidomics suggested that a reduction in IPCS2‐derived (G)IPCs causes a *pen* phenotype. Genetic analyses revealed that IPCS2 is involved in the PEN2/PEN3‐mediated defense against fungal intruders. Furthermore, confocal laser scanning microscopy (CLSM) indicated that IPCS2‐derived GIPCs at the TGN impact the polarized secretion of PEN3 to host‐pathogen contact sites. Our results uncover a role of complex sphingolipids in polar secretory trafficking of the immunity cargo PEN3 to sites of attempted fungal invasion.

## RESULTS

### Mutations in *IPCS2* result in enhanced cell entry success of non‐adapted powdery mildews

**Figure 1:**
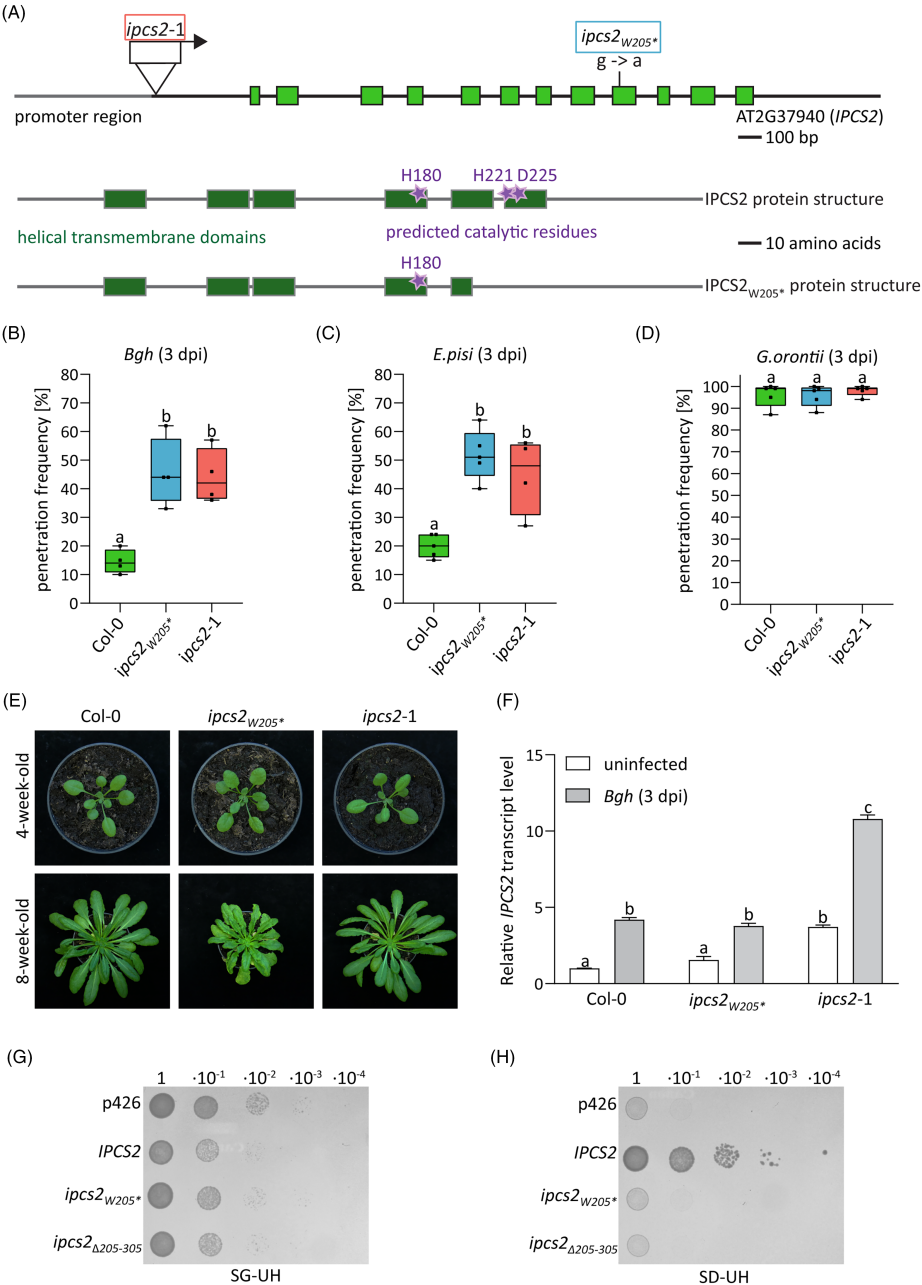
IPCS2 is involved in Arabidopsis cell entry control against non‐adapted pathogens. (A) Genomic organization of *IPCS2* including the promoter region and exon/intron structure, as well as the protein structure of IPCS2 and IPCS2_W205_*. The location of the ethyl methanesulfonate (EMS)‐induced premature stop codon insertion in the *ipcs2*_W205_* mutant is marked with a blue box, while the red box indicates the location and orientation of the T‐DNA insertion in the *ipcs2*‐1 mutant. (B–D) Penetration frequency of (B) *Bgh*, (C) *Erysiphe pisi* and (D) *Golovinomyces orontii* on wild‐type Col‐0, *ipcs2*_W205_*, and *ipcs2*‐1 mutant leaves at 3 days post infection (dpi). Individual box plots include whiskers representing minimum and maximum values. Letters show significant differences between genotypes (*n* = 4–5; one‐way ANOVA with Tukey's post‐hoc test; *P* < 0.05). Similar results were confirmed in at least two additional independent experiments. (E) Macroscopic phenotype of wild‐type Col‐0 and both *ipcs2* mutant variants after 4 or 8 weeks under short‐day conditions. (F) Uninfected (white bars) and *Bgh*‐infected (3 dpi; gray bars) leaves of wild‐type Col‐0, *ipcs2*_W205_* and *ipcs2*‐1 were analyzed by quantitative real‐time PCR. Presented *IPCS2* transcript levels are relative to the uninfected Col‐0 sample. The data represent the average ± standard error of the mean (*n* = 3–5). Letters show significant differences between genotypes and treatments (one‐way ANOVA with Tukey's post‐hoc test; *P* < 0.05). The experiment was repeated three times with similar results. (G, H) Complementation of yeast *YPH499‐HIS‐GAL1:AUR1* conditional mutant with the empty p426 vector (negative control), Arabidopsis wild‐type *IPCS2*, *IPCS2*_*W205*_, and *IPCS2*_*∆205–305*_. Expression of endogenous *AUR1* is induced in yeast cells grown on galactose‐containing media (SG‐UH; G) and suppressed on media supplemented with glucose instead of galactose (SD‐UH; H). The spotting assay for complementation was repeated twice using independent colonies, with similar results. Additionally, complementation phenotypes were confirmed by growth after streaking cells derived from four additional independent colonies recovered from transformation.

The *ipcs2*_W205_* mutant was isolated in a forward genetic screen for Arabidopsis plants that allow enhanced entry of the non‐adapted barley fungus *Blumeria graminis* f.sp. *hordei* (*Bgh*) into epidermal pavement cells (Figure S1). This mutation results in a premature stop codon, truncating IPCS2 at amino acid residue 205 (Figure 1A). As the truncation of IPCS2 results in the loss of one of two predicted catalytic motifs conserved among IPCSs (Huitema et al., 2004; Wang et al., 2008), the *ipcs2*_W205_* mutant likely represents a knock‐out and lacks the enzymatic activity of IPCS2 (Figure 1A). In the Arabidopsis ecotype Columbia 0 (Col‐0), only 15% ± 4% of germinated *Bgh* conidiospores successfully penetrate the plant cell wall at 3 days post infection (dpi), whereas the remaining attempts are blocked by the formation of the defense structure papilla (Figure 1B). In contrast, the *Bgh* penetration frequency increases to 46% ± 10% on the leaves of *ipcs2*_W205_* plants (Figure 1B). To confirm that the mutation in *IPCS2* causes the enhanced cell entry success of *Bgh* in this mutant, we analyzed another *ipcs2* mutant, which has a transfer DNA (T‐DNA) insertion 395 bp upstream of the translational start of *IPCS2* (Figure 1A). Like *ipcs2*_W205_*, *ipcs2*‐1 was more susceptible than the wild‐type Col‐0 to *Bgh* penetration (Figure 1B). Repeating this pathogenicity assay with the non‐adapted pea fungus *Erysiphe pisi* (*E. pisi*) yielded similar results, showing an increase of *E. pisi* entry rates up to a minimum of 45% ± 12% on both *ipcs2* mutants compared to the wild‐type Col‐0 (Figure 1C). Notably, the adapted powdery mildew *Golovinomyces orontii* (*G. orontii*) could grow on the *ipcs2* mutants at an equal level as on wild‐type leaves (Figure 1D), suggesting that IPCS2 function in Arabidopsis cell entry control is restricted to non‐adapted powdery mildews.

Macroscopic analyses revealed that the *ipcs2*_W205_* mutant exhibits a decreased rosette size and a spontaneous lesion phenotype at later growth stages, while no pleiotropic phenotype is visible for the *ipcs2*‐1 mutant (Figure 1E). To compare *IPCS2* transcript levels in both *ipcs2* mutants, we performed quantitative real‐time PCR (qPCR) with uninfected and *Bgh*‐infected (3 dpi) leaf samples of wild‐type Col‐0 and both *ipcs2* mutant alleles. Notably, this analysis revealed *Bgh*‐induced expression of *IPCS2* in the wild‐type Col‐0 (Figure 1F). The *IPCS2* transcript levels in uninfected and challenged *ipcs2*_W205_* mutant plants were similar to the wild‐type (Figure 1F), indicating that *IPCS2* transcript stability is not affected by the premature stop codon. In contrast, significantly elevated *IPCS2* expression was observed in uninfected *ipcs2*‐1 leaves and was even more upregulated upon pathogen attack (Figure 1F). Therefore, we concluded that the T‐DNA insertion in the promoter region of *IPCS2* induces overexpression in the *ipcs2*‐1 mutant via a yet unknown mechanism.

As the premature stop codon insertion in *IPCS2* does not affect transcript stability, we determined whether the truncated IPCS2_W205_* has residual enzymatic activity *in vivo* via a previously described yeast complementation assay (Mina et al., 2010). Thus, wild‐type *IPCS2* (Mina et al., 2010), *ipcs2*_W205_*, and the *ipcs2*_*∆205–305*_ variant, where the amino acids after the premature stop codon at position 205 are deleted, were cloned into a p426 expression vector (Mumberg et al., 1995; Sikorski & Hieter, 1989) under the control of the strong, constitutive *Saccharomyces cerevisiae GLYCERALDEHYDE‐3‐PHOSPHATE DEHYDROGENASE* (*GPD*) promoter. These constructs were transformed into the conditional mutant *YPH499–HIS3–GAL1:AUR1* (Denny et al., 2006), in which a galactose‐inducible promoter replaces the endogenous promoter of yeast *IPCS, AUREOBASIDIN A RESISTANCE1* (*AUR1*). Therefore, *AUR1* expression is induced by galactose‐containing medium, while the addition of glucose suppresses the expression. In contrast to the positive control wild‐type IPCS2, both IPCS2_W205_* mutant variants could not rescue the growth of the *YPH499‐HIS3‐GAL1:AUR1* strain (Figure 1G,H), indicating that the truncated IPCS2_W205_* protein has no residual enzymatic activity in yeast cells.

### Total ceramide content is enriched in *ipcs2*_*W205*_ *, while total (G)IPC content is reduced

**Figure 2:**
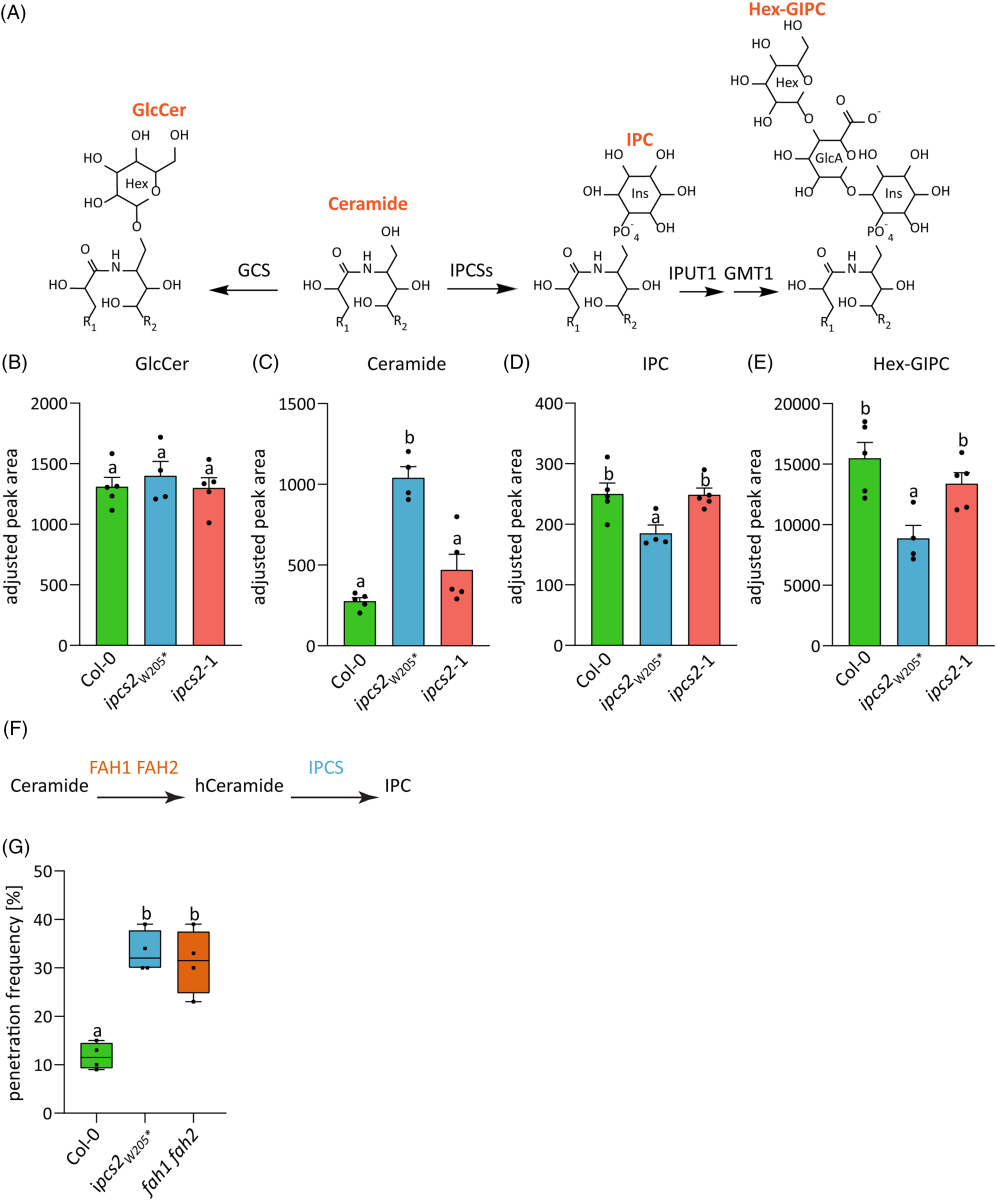
Sphingolipid profiles of Arabidopsis *ipcs2* mutants. (A) Different metabolic fates of ceramide. Transfer of a glucose residue to ceramide produces GlcCer. To generate GIPCs, an inositol phosphate is transferred onto the ceramide backbone by IPCS. Subsequently, a GlcA moiety is added by IPUT1, and GMT1 catalyzes mannosylation of the head group. R_1_ = FA; R_2_ = LCB. (B–E) Sphingolipid measurements of Arabidopsis rosettes for wild‐type Col‐0 and two *ipcs2* mutant alleles. Values are individual MRM peak areas normalized to the total fatty acid methyl ester (FAME) quantity and mg dry weight per sample. Bars represent averages of four to five biological replicates; error bars are standard error of the mean. Letters indicate significance at *P* < 0.05 determined by a one‐way ANOVA with Tukey's post‐hoc test. Similar results were obtained in an independent experiment. (F) The substrate production of IPCS2 is facilitated by FAH1 and FAH2, which hydroxylate ceramides. (G) Box plots demonstrate the *Bgh* penetration frequency on wild‐type Col‐0, *ipcs2*_W205_* and *fah1 fah2* leaves at 3 days post infection (dpi). Whiskers include minimum and maximum values. Letters show significant differences between genotypes (*n* = 4–5; one‐way ANOVA with Tukey's post hoc test; *P* < 0.05). Similar results were obtained in two additional independent experiments.

To further assess the effect of the *ipcs2*_W205_* mutation and the T‐DNA insertion in *ipcs2*‐1 *in planta*, we determined their sphingolipid profiles using targeted UPLC‐nanoESI‐MS/MS analysis. We measured ceramides, glucosylceramide (GlcCer), IPC, and different classes of GIPCs. Briefly, the modification of ceramide with a glucose residue by glucosylceramide synthase (GCS) generates GlcCer (Leipelt et al., 2001; Melser et al., 2010) (Figure 2A). To produce GIPCs, IPCSs catalyze the addition of a head group derived from PI to ceramides for the production of the intermediate IPC (Mina et al., 2010) (Figure 2A). The next step involves the addition of a glucuronic acid (GlcA) moiety via an α‐(1,4)‐linkage to IPC, catalyzed by the inositol phosphorylceramide glucuronosyltransferase 1 (IPUT1) (Rennie et al., 2014) (Figure 2A). Further production of Hex‐GIPC requires the GIPC mannosyl transferase 1 (GMT1) (Figure 2A), while HexNAc‐GIPC synthesis depends on glucosamine inositol phosphorylceramide transferase 1 (GINT1) (Fang et al., 2016; Ishikawa et al., 2018). In comparison to the wild‐type Col‐0, ceramide levels were significantly enriched in *ipcs2*_W205_*, but not in *ipcs2*‐1, whereas the GlcCer content was unaffected by the mutations in *IPCS2* (Figure 2B,C). Notably, IPC and Hex‐GIPC levels were significantly reduced in *ipcs2*_W205_*, while *ipcs2*‐1 showed similar levels as the wild‐type Col‐0 (Figure 2D,E). These results suggest that the premature stop codon insertion in *ipcs2*_W205_* leads to a knock‐out mutation and loss of enzymatic activity of IPCS2, resulting in an accumulation of ceramide and a reduction in IPC as well as Hex‐GIPC levels. In contrast, the sphingolipid profile of *ipcs2*‐1 was wild‐type‐like, suggesting that the T‐DNA insertion in the *IPCS2* promoter region does not impact the biochemical function of IPCS2 and sphingolipid homeostasis. Further investigations are necessary to clarify the underlying mechanism that causes the compromised penetration resistance in *ipcs2*‐1. The remainder of the present study, however, is focused on the *ipcs2*_W205_* mutant.

As the IPC and Hex‐GIPC content levels in *ipcs2*_W205_* were significantly reduced but not fully abolished, we suspected that the IPCS2 homologs IPCS1 and IPCS3 may contribute to the synthesis of IPC in this mutant. Based on expression profiles, *IPCS2* is the most highly expressed *IPCS* isoform in all tissues, while *IPCS1* exhibits weak constitutive expression (0.05% of *IPCS2* levels in rosette leaves) (Mina et al., 2010). In contrast, *IPCS3* has high constitutive expression in stems and flowers and is inferred to have a specialized role in these tissues (Mina et al., 2010). As a common problem with strong alteration of GIPC levels, double knockout *ipcs1 ipcs2* mutants could not be analyzed due to seedling lethality (Ito et al., 2021; Tartaglio et al., 2017). Therefore, we analyzed the previously described Arabidopsis inducible *IPCS1;2* ^*amiRNA*^ knockdown line (Ito et al., 2021). In contrast to the *ipcs2*_W205_* mutant, the *Bgh* penetration frequency was only slightly increased for the *IPCS1;2* ^*amiRNA*^ knockdown line after β‐Estradiol (E2) treatment, but not significantly different from the wild‐type Col‐0 (Figure S2A). This attenuated effect might be due to the E2‐induced decrease in *IPCS1* and *IPCS2* transcript levels of only 54% and 48%, respectively, in the *IPCS1;2* ^*amiRNA*^ line (Figure S2B,C). This relatively mild phenotype compared to the *ipcs2*_W205_* mutant allele suggests that IPCS2, and not IPCS1, has a predominant role in cell entry control. This was further supported by the finding that the *ipcs1* single knockout exhibits a wild‐type‐like *Bgh* infection phenotype (Figure S2D). Interestingly, similar to *IPCS2*, *IPCS1* transcript levels were increased by *Bgh* infection in the wild‐type Col‐0 and were even more upregulated in the *ipcs2*_W205_* mutant (Figure S2E). Taken together, our results suggest that IPCS2 is the major contributor to cell entry control, while in the absence of IPCS2, *IPCS1* is likely upregulated to partially complement this function.

### IPCS2‐derived (G)IPCs are required for Arabidopsis pathogen entry control

To further assess whether the reduction of IPC and Hex‐GIPC levels in *ipcs2*_W205_* causes the enhanced cell entry success of non‐adapted powdery mildews, we tested the double mutant of FATTY ACID HYDROXYLASE 1 and 2 (FAH1/2), which hydroxylate the α position of the fatty acid moiety in ceramides, thereby contributing to the production of the IPCS2 substrate (König et al., 2012, 2022; Nagano et al., 2012) (Figure 2F). Similar to *ipcs2*_W205_*, the penetration frequency of *Bgh* was increased up to 31% ± 6% on *fah1 fah2* leaves compared to the wild‐type Col‐0 (Figure 2G). This supports the hypothesis that the products of IPCS2 are required for cell entry control, rather than a moonlighting function of the IPCS enzyme.

### IPCS2 is involved in the Arabidopsis PEN2/PEN3‐dependent pathogen entry control pathway

**Figure 3:**
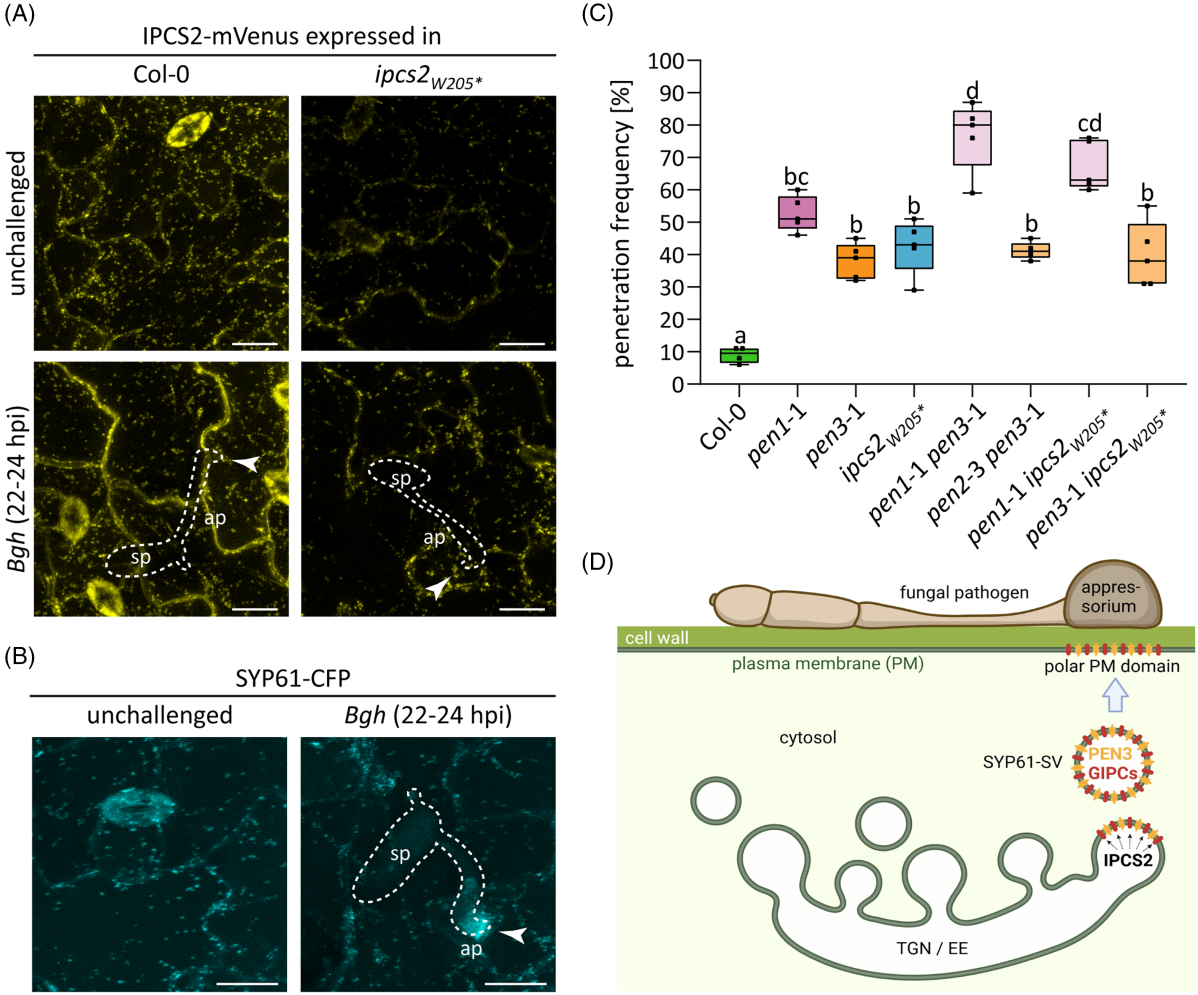
IPCS2 is part of the PEN2/PEN3 pathway. (A) Stable transgenic Col‐0 and *ipcs2*_W205_* plants expressing *pUBQ10:IPCS2‐mVenus* were analyzed by CLSM. Maximum z‐projections of CLSM images from unchallenged or *Bgh*‐infected (22–24 hours post infection [hpi]) leaf epidermal cells are presented. *Bgh*‐plant interaction sites are marked with a white arrowhead. White lines indicate the fungal spore with appressorium formation. Ap, appressorium; sp, spore. Scale Bar = 20 μm. (B) Maximum z‐projections of CLSM images from unchallenged or *Bgh*‐infected (22–24 hpi) leaf epidermal cells of Col‐0 plants stably expressing *pSYP61:SYP61‐CFP*. Scale bar = 10 μm. (C) Box plots illustrate the *Bgh* penetration frequency on the indicated single and double mutants at 3 days post infection. Whiskers represent minimum and maximum values, while letters show significant differences between genotypes (*n* = 5; one‐way ANOVA with Tukey's post‐hoc test; *P* < 0.05). (D) Model illustrating IPCS2‐dependent production of GIPCs, which are incorporated into SYP61‐SVs and likely shuttle defense cargo such as PEN3 to polar domains underneath sites of attempted fungal invasion. Model created with Biorender.com. All results shown in this figure were replicated in two additional independent experiments.

In steady‐state, IPCS2 was found to co‐localize with the SYNTAXIN OF PLANTS 61‐secretory vesicle (SYP61‐SV) subdomain of the TGN (Ito et al., 2021). The next step was to track the subcellular behavior of IPCS2 and the associated (G)IPC production upon pathogen attack. Therefore, stable transgenic Col‐0 and *ipcs2*_W205_* plants expressing *pUBQ:IPCS2‐mVenus* were generated. Infection phenotype analyses confirmed that IPCS2‐mVenus can restore the *Bgh* penetration rates on *ipcs2*_W205_* mutant leaves to wild‐type levels (Figure S3A). CLSM studies revealed that this functional IPCS2‐mVenus fusion protein does not accumulate at *Bgh* infection sites in neither wild‐type nor *ipcs2*_W205_* mutant plants (Figure 3A). This suggests that IPCS2 is not subjected to pathogen‐induced polarization to infection sites, and rather stays at the TGN for (G)IPC production during pathogen attack.

Emerging evidence suggests that SYP61‐SVs play a crucial role in the polar transport of GIPC‐enriched cargo from the TGN to polar domains of the PM (Tapken & Murphy, 2015; Wattelet‐Boyer et al., 2016). CLSM analysis of a transgenic SYP61‐CFP marker line revealed focal accumulation of SYP61‐CFP at the tip of the *Bgh* appressorium (Figure 3B), indicating that SYP61‐SVs are involved in the polarized secretion of defense material to domains underneath sites of fungal invasion attempts. As previous proteomic studies of the SYP61‐SVs/TGN immuno‐purified fractions revealed PEN1 and PEN3 as cargo of SYP61‐SVs (Ito et al., 2021), we hypothesized that IPCS2‐derived GIPCs could facilitate the shuttling of PEN1 and/or PEN3 to polar domains at sites of attempted fungal invasion. As genetic analyses revealed that PEN1 and PEN2/PEN3 act in different pathways (Lipka et al., 2005; Stein et al., 2006), we performed the same approach with *ipcs2*_W205_* mutants to clarify in which pathway GIPCs are involved. To generate double mutants, *ipcs2*_W205_* was crossed to *pen1*‐1 and *pen3*‐1 single mutants. The *Bgh* penetration rate of the control *pen1*‐1 *pen3*‐1 double mutant was elevated in comparison to the single mutants, while the cell entry success of *Bgh* on *pen2*‐3 *pen3*‐1 leaves was similar to the single mutants (Figure 3C), confirming previous findings (Johansson et al., 2014; Stein et al., 2006). In contrast to *pen3*‐1 *ipcs2*_W205_* double mutants, the *Bgh* penetration rate was increased for *pen1*‐1 *ipcs2*_W205_* in comparison to the single mutants (Figure 3C), indicating that IPCS2 is more likely involved in the PEN2/PEN3‐dependent pathway. Thus, we propose a model in which IPCS2 strongly produces (G)IPCs at the TGN upon powdery mildew attack (Figure 3D). These GIPCs are incorporated into SYP61‐SVs for shuttling PEN3 to polar domains at sites of attempted fungal invasion (Figure 3D). GIPCs were previously reported to mediate PI4P consumption at the TGN and subsequent polar sorting of the auxin efflux carrier PIN2, via activation of phosphoinositide‐specific phospholipases C (PI‐PLC) (Ito et al., 2021). We hypothesized that PI4P consumption could also be a mechanism of IPCS2‐mediated polar recruitment of immunity cargo such as PEN3 to sites of attempted fungal invasion. Upon infection with *Bgh*, PI4P accumulated at the tip of the appressorium (Figure S4). However, as PI4P signals were extremely weak in leaf tissue, we failed to correlate any change in PI4P distribution at *Bgh* penetration sites with loss of IPCS2 function in the *ipcs2*_W205_* mutant background.

### Focal accumulation of PEN3 at sites of attempted fungal invasion requires IPCS2 activity

**Figure 4:**
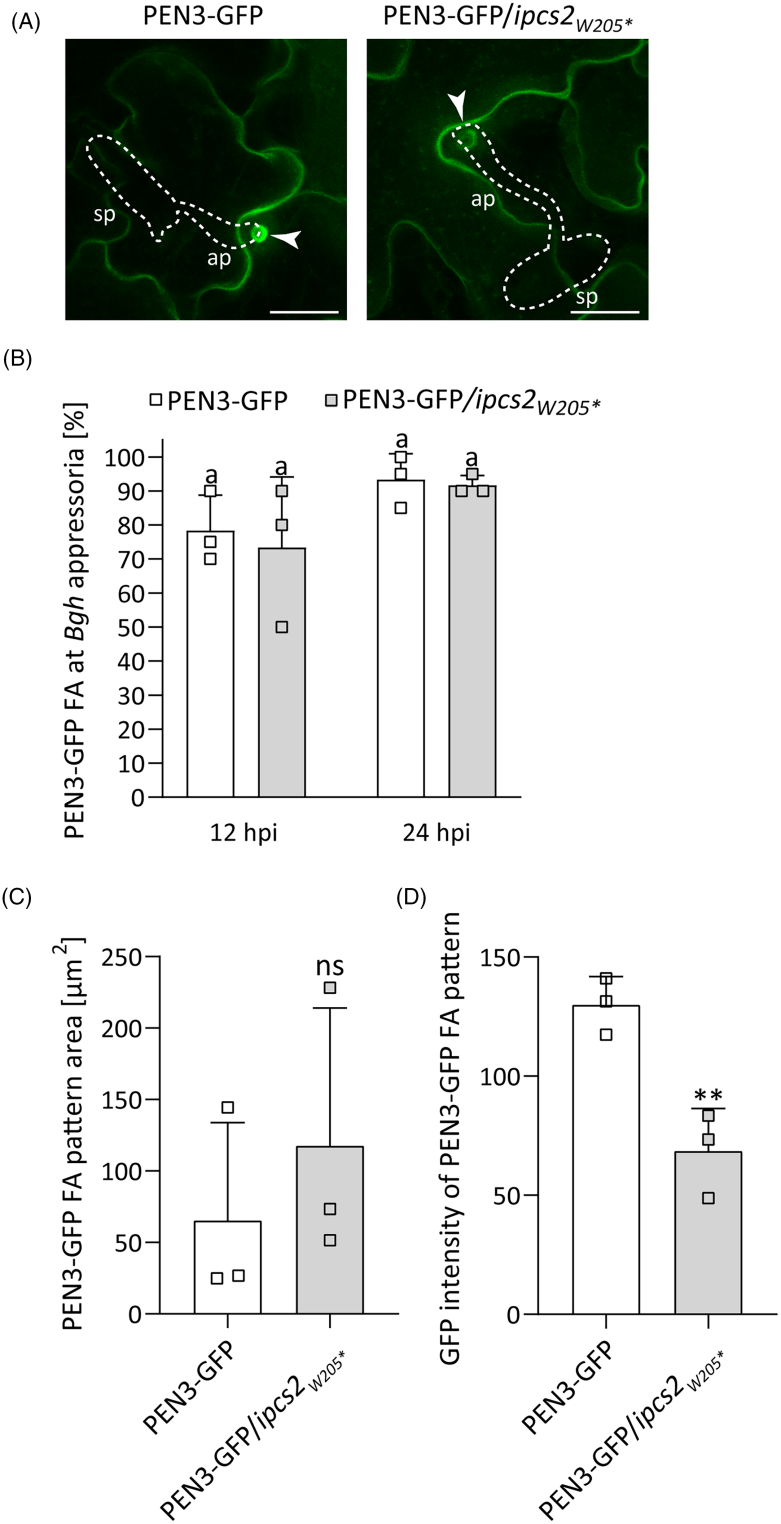
IPCS2 enzyme activity plays a crucial role in the polarized secretion of PEN3 to sites of attempted fungal invasion. (A) Maximum z‐projections of CLSM images reveal focal accumulation (FA) of PEN3‐GFP at *Bgh* (24 hours post infection [hpi]) penetration sites (arrowheads) in the mother plant (left panel) or in the *ipcs2*_W205_* mutant background (right panel). White lines indicate the fungal spore with appressorium formation. Ap, appressorium; sp, spore. Scale Bar = 20 μm. (B) Time‐course evaluation of PEN3‐GFP FA at plant‐*Bgh* contact sites at 12 hpi and 24 hpi in the mother plant (white bars) or in the *ipcs2*_W205_* mutant background (gray bars). PEN3‐GFP FA patterns were scored at the tip of 20 *Bgh* appressoria. Bars represent averages of three biological replicates ± standard deviation. Letters indicate significance at *P* < 0.05 determined by one‐way ANOVA with Tukey's post‐hoc test. (C, D) Quantification of (C) the area (μm^2^) or (D) the GFP intensity of PEN3‐GFP FA patterns at the tip of 17–20 *Bgh* appressoria (24 hpi) in the mother plant (white bars) or in the *ipcs2*_W205_* mutant background (gray bars). Bars show the average and error bars represent standard deviation (*n* = 3). Significant differences to the mother plant were calculated with a Student's *t*‐test (ns = non‐significant; **P < 0.0077). All experiments shown in this figure were repeated three times with similar outcomes.

To test the hypothesis that IPCS2‐derived GIPCs are required for polar transport of PEN3 to sites of attempted fungal invasion, we crossed *ipcs2*_W205_* with the published transgenic protein marker line *pPEN3:PEN3‐GFP* (Stein et al., 2006). In CLSM analyses, PEN3‐GFP fluorescence intensity of focal accumulation (FA) patterns at *Bgh* appressoria appeared to be reduced in the *ipcs2*_W205_* mutant background compared to the mother plant at 24 hours post infection (hpi) (Figure 4A). To evaluate whether this reduction of PEN3 FA at *Bgh* penetration sites is caused by delayed recruitment, we performed a time course experiment. Therefore, the frequency of PEN3‐GFP FA patterns at *Bgh* penetration sites was assessed at 12 and 24 hpi. The frequency of PEN3‐GFP FA at *Bgh* appressoria was similar between the control line and PEN3‐GFP in *ipcs2*_W205_* at both time points (Figure 4B). Thus, we hypothesized that dysfunction of IPCS2 enzymatic activity could impair the spatial rather than temporal recruitment of PEN3‐GFP to sites of attempted fungal invasion, or perhaps the extent and intensity of PEN3 aggregation. To test this idea, the area and mean intensity of these PEN3‐GFP FA patterns in the mother plant and the *ipcs2*_W205_* mutant background were quantified at 24 hpi. This quantification analysis demonstrated that the area of PEN3‐GFP FA patterns at *Bgh* appressoria is unaffected by IPCS2 dysfunction, whereas the fluorescence intensity was significantly reduced in the *ipcs2*_W205_* mutant by 53% ± 11% relative to the mother plant (Figure 4C,D). In contrast, the recruitment of callose as a molecular marker of papillae was unaffected in the *ipcs2*_W205_* mutant (Figure S5). Taken together, these results suggest that IPCS2 enzymatic activity is involved in the specialized delivery of PEN3 to host–powdery mildew contact sites.

## DISCUSSION

IPCS2 is primarily known for its involvement in the synthesis of complex sphingolipids (Mina et al., 2010). Here, we demonstrate that genetic interference of *IPCS2* causes enhanced cell entry success of non‐adapted powdery mildews, indicating a role of IPCS2 in penetration resistance. In this study, the novel *ipcs2*_W205_* mutant was identified, in which a premature stop codon results in loss of enzymatic activity in yeast cells and *in planta*. Our sphingolipidomic analysis revealed that compromised IPCS2 function results in ceramide accumulation and reduction of (G)IPC content, confirming published findings (Ito et al., 2021).

*IPCS2* was previously recovered from an enhancer screen for RESISTANCE TO POWDERY MILDEW 8 (RPW8), which is involved in post‐penetration resistance to several powdery mildew species (Wang et al., 2008; Xiao et al., 2001). Notably, the study of Wang et al. (2008) demonstrated that increased ceramide levels in the absence of IPCS2 induce salicylic acid (SA) accumulation, resulting in a lesion phenotype. In alignment with these findings, the *ipcs2*_W205_* mutant exhibited spontaneous cell death at later growth stages, which was abolished when SA synthesis (SALICYLIC ACID INDUCTION DEFICIENT 2 [SID2]) and signaling pathways were blocked (ENHANCED DISEASE SUSCEPTIBILITY 1, EDS1; PHYTOALEXIN‐DEFICIENT, PAD4) by crossing the *ipcs2*_W205_* mutant to the triple *eds1*‐2/*pad4*‐1/*sid2*‐2 (*eps*) mutant (Zhang et al., 2018) (Figure S6). Furthermore, constitutive expression of IPCS2‐mVenus under the strong *UBQ10* promoter in Col‐0 and in the *ipcs2*_W205_* mutant resulted in spontaneous cell death (Figure S3B), indicating that not only disruption but also overexpression of IPCS2 causes lesions. This supports the current model based on several publications (Bi et al., 2014; König et al., 2012; Simanshu et al., 2014; Zeng et al., 2021; Zeng & Yao, 2022) that normal and controlled sphingolipid metabolism plays a crucial role in SA homeostasis, and that ceramides act as signaling lipids that induce PCD via SA. Accumulation of free ceramides or hydroxyceramides has been observed in several mutants specifically impaired in GIPC headgroup assembly, correlating with SA accumulation and the development of necrotic lesions reminiscent of PCD associated with the hypersensitive response (Mortimer et al., 2013; Tartaglio et al., 2017; Wang et al., 2008). In contrast to our finding that IPCS2 does not affect susceptibility to *G. orontii*, previous research on *gonst2* demonstrated increased resistance to this adapted powdery mildew (Jing et al., 2021). The *gonst2* mutant lacks a functional nucleotide sugar transporter (GOLGI LOCALIZED NUCLEOTIDE SUGAR TRANSPORTER 2), which is presumed to play a role in GIPC headgroup assembly. However, single mutants do not exhibit substantial GIPC depletion. This indicates that additional factors, or potentially cell‐type‐specific metabolic responses to *G. orontii*, may contribute to the observed enhanced resistance.

Synthesis of GIPCs with specific headgroups can also impact interactions between plant and microbial cells. For example, GIPCs are ligands of necrotizing cytolysins produced by diverse oomycete, bacterial, and fungal pathogens. The degree of GIPC headgroup glycosylation, which varies among plant lineages, determines whether the necrotizing cytolysin can simultaneously bind and permeate the host cell plasma membrane (Lenarčič et al., 2017). Further, the local, preferential synthesis of HexNAc‐GIPCs over Hex‐GIPCs in symbiosome and periarbuscular membranes was demonstrated to be essential for supporting symbiotic interactions of *Medicago truncatula* with nodulating rhizobia and arbuscular mycorrhiza, respectively (Moore et al., 2021). Therefore, GIPC synthesis has the potential to impact plant–microbe interactions via three distinct mechanisms: (1) by regulating ceramide levels that trigger post‐penetration defenses, (2) via interplay between microorganisms and host cell plasma membranes accumulating specific GIPC headgroups, and (3) by contributing to polar sorting of membranes to sites of pathogen ingress, as investigated here.

In addition to the *ipcs2*_W205_* mutant, we analyzed another *ipcs2* mutant with defective penetration resistance, *ipcs2‐*1, which has a T‐DNA insertion at the end of the promoter region of *IPCS2*. qPCR revealed that *IPCS2* transcript levels are upregulated in uninfected *ipcs2*‐1 plants, suggesting that the T‐DNA insertion induces an increase in expression either by disrupting negative regulatory sequences or by insertion of activating sequences encoded by the T‐DNA. However, *ipcs2*‐1 mutants exhibit a wild‐type‐like macroscopic phenotype, while Col‐0 plants stably expressing IPCS2‐mVenus from a strong constitutive promoter showed a lesion phenotype (Figure S3B). This suggests that IPCS2 protein may not constitutively hyperaccumulate in the *ipcs2*‐1 mutant, which is supported by a wild‐type‐like sphingolipid profile. This is in alignment with the findings of a previous study reporting only subtle differences between wild‐type Col‐0 and *ipcs2*‐1 in a sphingolipidomic analysis (Yan et al., 2019). However, the qPCR and sphingolipidomic data are based on a single leaf or whole rosette, respectively. Therefore, different cell type‐specific expression of *IPCS2*, cell type‐specific activity changes, or different subcellular behavior upon pathogen attack cannot be ruled out. As powdery mildew infection is restricted to the epidermis (Hückelhoven & Panstruga, 2011), it would be interesting to analyze the epidermal expression profile of *IPCS2* in this T‐DNA insertional mutant. Furthermore, *ipcs2*_W205_* mutant plants could be generated that stably express native IPCS2 tagged to mVenus from the *ipcs2*‐1 promoter. This would allow subcellular tracking of the IPCS2 mutant variant in the epidermis upon pathogen attack.

Furthermore, *IPCS2* transcript levels were found to be elevated upon *Bgh* infection, confirming previous findings showing that *IPCS2* is upregulated by non‐adapted powdery mildews (Nishimura et al., 2003; Stein et al., 2006; Wang et al., 2008). This pathogen‐induced transcription was also observed in the *ipcs2*‐1 mutant. In transgenic lines stably expressing IPCS2‐mVenus under the control of a constitutive promoter in the wild‐type or *ipcs2*_W205_* mutant background, IPCS2‐mVenus protein levels were increased upon *Bgh* infection in Western blot analyses (Figure S3C). This suggests a post‐transcriptional stabilization of *IPCS2* RNA and/or IPCS2 protein upon pathogen attack, which presumably serves to increase GIPC production to terminate fungal ingress.

Mutant analyses of other components functioning upstream or downstream of IPCS2 lend further support to the idea that IPCS2‐derived GIPCs are required for cell entry control against non‐adapted fungal pathogens. Previous findings provided evidence that GIPCs contribute to the polar sorting of the auxin efflux transporter PIN2 without affecting endocytosis or recycling (Markham et al., 2011; Wattelet‐Boyer et al., 2016). Here, we demonstrate that IPCS2‐derived GIPCs are also required for polarized secretion of PEN3 to sites of attempted fungal invasion. In contrast, genetic analyses excluded IPCS2 function from the PEN1‐dependent pathway and time‐course experiments revealed that callose deposition at papillae is IPCS2‐independent. As polar transport of the callose synthase POWDERY MILDEW RESISTANT 4 (PMR4)/GLUCAN SYNTHASE‐LIKE 5 (GSL5) from the TGN to sites of attempted fungal invasion presumably depends on the PEN1 pathway (Assaad et al., 2004; Nielsen et al., 2012; Qin et al., 2021; Rubiato et al., 2022), we infer that IPCS2 acts in a specialized polar trafficking pathway for PEN3 recruitment. The transport model recently proposed by Nielsen (2024) suggests that recycled PEN1 and PEN3 overlap at the TGN upon pathogen infection, which is followed by splitting into distinct pathways for recruitment of both PEN proteins to sites of attempted fungal invasion. As PEN1, PMR4, PEN3, and IPCS2 were reported to be localized to the SYP61‐SV subdomain of the TGN (Drakakaki et al., 2012; Ito et al., 2021), it is likely that this represents the shared TGN compartment after recycling. In this study, we demonstrate that upon pathogen attack, IPCS2‐mVenus remains at the TGN, while SYP61‐CFP accumulates at sites of attempted fungal invasion. It is therefore reasonable to postulate that IPCS2‐dependent sorting processes of PEN3 may occur in the TGN, possibly followed by delivery via SYP61‐labeled vesicles to fungal entry sites. However, it remains to be shown at which particular step and in which exact (post‐)TGN compartment recycling and polar secretion is controlled by IPCS2‐dependent GIPCs and to what extent specialized subpopulations of endosomes, multi‐vesicular bodies, or exosomes may be involved in the distinct transport routes of PEN1 and PEN3. IPCS2‐derived GIPC association with other lipids or proteins could potentially direct PEN3 as a specific cargo for transport to pathogen‐induced polar domains, while PEN1 is excluded and follows a sphingolipid‐independent secretory mechanism. Taken together, we provide evidence for a mechanistic link between IPCS2‐derived complex sphingolipids and proper sorting of PEN3 to sites of attempted fungal invasion, uncovering a GIPC‐dependent polar secretory trafficking pathway in Arabidopsis cell entry control.

## MATERIALS AND METHODS

### Plant material and growth conditions

The *Arabidopsis thaliana* ecotype Columbia (Col‐0) was used as the wild‐type. The following previously characterized single/double/triple mutants were analyzed: *ipcs1* (Ito et al., 2021), *fah1 fah2* (König et al., 2022), *pen1*‐1 (Collins et al., 2003), *pen3*‐1, *pen1*‐1 *pen3*‐1, *pen2*‐3 *pen3*‐1 (Stein et al., 2006) and *eps* (*eds1*‐2/*pad4*‐1/*sid2*‐2) (Zhang et al., 2018). The seeds of the T‐DNA insertion mutants *ipcs2*‐1 (SALK_206784) and *ugnt1*‐4 (SALK_075727) were obtained from the Nottingham Arabidopsis Stock Centre, and genomic insertions of the T‐DNAs were confirmed by PCR. All primers used in this study are listed in Table S1. Double and quadruple mutants of *ipcs2*_W205_* were generated by crossing with the respective single/triple mutant, and homozygous mutants were confirmed by PCR and Sanger sequencing. The following previously described Arabidopsis transgenic fluorescent protein marker lines were used: *pSYP61:SYP61‐CFP* (Robert et al., 2008), *pPEN3:PEN3‐GFP* (Stein et al., 2006) and *pUBQ10:mCITRINE‐3 × PH* ^*FAPP1*^ (Simon et al., 2014). Additionally, the recently established β‐Estradiol‐inducible *IPCS1;2* artificial microRNA line (Ito et al., 2021) was used for a pathogenicity assay.

Arabidopsis plants were grown under short‐day conditions (8‐h photoperiod, 22°C [day]/18°C (night), 65% relative humidity and a light intensity of approximately 150 μmol m^−2^ sec^−1^) and used for experiments after 4 weeks.

### Generation of a pUBQ10:IPCS2‐mVenus line

The previously described *pUBQ10:IPCS2‐mVenus* construct was designed using the multisite gateway system (Esnay, 2020). The construct was confirmed by Sanger sequencing, transformed into *Agrobacterium tumefaciens* C1C58, and used for stable transformation of Col‐0 and *ipcs2*_W205_* plants according to the floral dip method (Clough & Bent, 1998). To select transgenic plants, soil‐grown T1 seedlings were sprayed with BASTA (Bayer CropScience).

### Arabidopsis inoculation and powdery mildew pathogenicity assay

*Blumeria graminis* f.sp. *hordei* isolate K1 conidiospores were generated on *Hordeum vulgare* cv Lottie for 10 to 14 days, *Erysiphe pisi* (*E. pisi*) spores were produced on *Pisum sativum* (kleine Rheinländerin) for 14 days, and *Golovinomyces orontii* (*G. orontii*) was cultivated on 4‐week‐old Arabidopsis plants for 10–14 days. For pathogenicity assays, 4‐week‐old Arabidopsis plants were infected with *Bgh*, *E. pisi*, and *G. orontii*. After 22–24 hpi, plants were used for CLSM and after 3 dpi for screening and quantification of fungal invasive growth rates. Visualization of papillae and haustoria formation as well as epidermal cell death was performed according to the established method by Lipka et al. (2005). For calculation of the penetration frequency (number of haustoria + epidermal cell death), 100 interaction sites per leaf of three to five individual plants per genotype were scored.

### Arabidopsis *pen* mutant screen and mapping of *ipcs2*_*W205*_ _*_

M6 populations of Col‐0 homozygous EMS mutant (HEM) lines (Capilla‐Perez et al., 2018) were obtained from the Versailles Arabidopsis stock center. Plants were inoculated with *Bgh* and 3 days later screened for enhanced autofluorescence, indicative of cell death due to fungal entry, using a GFP1 filter set (excitation filter 425/60 nm; dichroic mirror 480 nm; Leica Microsystems) on a Leica M165 FC fluorescence‐stereomicroscope. For mapping, a previously reported approach (Hartwig et al., 2012) was adapted. Thus, the *ipcs2*_W205_* mutant was backcrossed to Col‐0. Approximately 380 F2 pLants were screened for enhanced cell death rates. Twenty‐eight plants with a clear *pen* phenotype were pooled and subjected to 150‐bp paired‐end Illumina sequencing. The resulting reads were mapped to the Col‐0 TAIR10 genome by using the CLC genomics workbench software and single nucleotide polymorphisms (SNPs) with a frequency of 20% or higher were called. SNPs present in Col‐0 were subtracted from SNPs found in *ipcs2*_W205_*. The frequency of the remaining SNPs was plotted against their chromosomal position. The observed frequency peak on chromosome 2 contained a SNP resulting in a premature stop codon (W205*) in *IPCS2* (AT2G37940) (Figure S1). A complementation analysis confirmed this polymorphism as the causative mutation.

### Inhibitor treatment

For amiRNA induction, 4‐week‐old plants of the *IPCS1;2* ^*amiRNA*^ line as well as the control plants were sprayed with 25 μM β‐Estradiol (Sigma‐Aldrich) and used for infection experiments after 48 h.

### Quantitative RT‐PCR

RNA was extracted from Arabidopsis leaves using the innuPREP Plant RNA Kit (Innuscreen). cDNA was generated with RevertAid H Minus m‐MuLV Reverse Transcriptase (Thermo Fisher Scientific). According to the manufacturer's recommendations, SsoFast EvaGreen Supermix was used to perform qPCR in a CFX96 Real‐Time PCR System (BioRad). The amplification protocol started with a 30 sec initial denaturation at 95°C and was followed by 45 cycles of 5 sec at 95°C and 55°C for 10 sec. Single product amplification was checked by recording the melting curves. For each transcript, dilution series of pooled cDNA were performed under the same conditions for primer efficiency calculations. *IPCS2* was amplified using primers JM231 and JM280 (Table S1). *IPCS1* was amplified using the published primers by Ito et al. (2021). *IPCS1* and *IPCS2* expression levels were normalized to *ACTIN8* (amplified with primers EP223 and EP224; Table S1).

### Yeast complementation assay

The premature stop codon insertion (W205*) or deletion of the remaining amino acids following W205 in IPCS2 was inserted in the previously published p426‐*AtIPCS2* expression plasmid (Haslam et al., 2024; Mina et al., 2010) using the Q5 Site‐Directed Mutagenesis Kit (New England Biolabs). All primers used for cloning are listed in Table S1. The plasmid sequences were confirmed by Sanger sequencing. The previously reported LiAc/SS carrier DNA/PEG method was used to transform the different expression plasmids in parallel into *YPH499‐HIS3‐GAL1*_*pro*_:*AUR1* cell aliquots (Gietz & Schiestl, 2007). Uptake of the correct plasmid was verified by colony PCR and Sanger sequencing for all colonies used for the complementation assay. Yeast strains were cultivated in or on synthetic complete dropout medium (Gietz & Woods, 2002) with(out) the appropriate nutrients and carbohydrate supplementation.

### Sphingolipidomics

Microsomes were enriched from 10 mg of lyophilized Arabidopsis rosette tissue by following a previously described protocol (Abas & Luschnig, 2010). The established monophasic extraction method using isopropanol:hexane:water (60:26:14, v/v/v) (Markham et al., 2006) was performed for lipid extraction. Half of the total lipid extract volume was taken for methylamine treatment to hydrolyze glycerolipids. This allowed a cleaner background by removing false positive signals from the sphingolipid analysis and was based on the protocol described by Markham (2013). The sphingolipid analysis followed the established protocol by Herrfurth et al. (2021). In short, methylamine‐treated sphingolipids were separated by an ACQUITY UPLC system (Waters Corp.) supplied with an HSS T3 silica‐based reversed‐phase C18 column (100 mm × 1 mm, 1.8 μL; Waters Corp.). Ionization was performed by a chip‐based nano‐electrospray using TriVersa Nanomate (Advion BioScience) with 5 μm internal diameter nozzles. An AB Sciex 6500 QTRAP tandem mass spectrometer (AB Sciex) was used for analysis, which was operated in positive ionization mode of the nanomate and in multiple reaction monitoring (MRM) mode of the mass spectrometer. A 2 μL sample was injected by an autosampler set at 18°C. The flow rate of the sample separation was 0.1 mL min^−1^. Solvent A contained methanol‐20 mM ammonium acetate (3:7, v/v) with 0.1% acetic acid (v/v), while solvent B was composed of tetrahydrofuran:methanol:20 mM ammonium acetate (6:3:1, v/v/v) with 0.1% acetic acid (v/v). Sphingolipids were separated by a linear solvent gradient, starting from 65% B for 2 min, which increased to 100% B in 8 min, holding for 2 min and re‐equilibrating to the starting conditions in 4 min. For the targeted analysis, ceramides and glycosylceramides were detected as [M + H]^+^ (precursor ions) and by the dehydrated LCB fragments (product ions) whereas glycosyl inositol phosphorylceramides were detected as [M + NH_4_]^+^ (precursor ions) and by the ceramide backbone (product ions).

A 20% aliquot was used for methyl esterification by sulfuric acid‐catalyzed methanolysis described in Haslam et al. (2024) for fatty acid methyl ester (FAME) normalization. Gas chromatography with flame ionization detection (GC‐FID) was employed for total fatty acid quantification. For relative comparisons among genotypes, the LC–MS MRM peak areas of individual lipid species were normalized to the total fatty acid content of each sample.

### Confocal microscopy

Confocal microscopy was performed on a Leica TCS SP8 Falcon system equipped with a HyD SMD detector. mVenus and mCitrine fluorescent proteins were excited with a pulsed white light laser at 514 nm, and fluorescence emission was recorded between 530 and 600 nm. The excitation wavelength for GFP was 488 nm, and its fluorescence emission was detected between 500 and 540 nm. To exclude chloroplast autofluorescence signals from images, a fluorescence lifetime gate was set between 0.4 and 6 ns. The TGN marker SYP61‐CFP was excited using a 405 nm Diode laser, and fluorescence emission was captured between 460 and 500 nm. The Leica LASX 3.5.5 software or the LIF Projector plugin in Fiji (‐ImageJ v1.49m) was used to export z‐stacks (12 consecutive focal plane images with a distance of 1 μm) as maximum projections. Brightness/contrast was adjusted with Adobe Photoshop CS5.

### Time‐course experiment of pathogen‐induced PEN3 and callose accumulation

To evaluate the subcellular behavior of PEN3 in the *ipcs2*_W205_* mutant, this mutant was crossed with the published stable transgenic *pPEN3:PEN3‐GFP* line (Stein et al., 2006). Homozygous F3 plants and the mother plant were inoculated with *Bgh* and analyzed between 12 and 24 hpi via CLSM. Twenty *Bgh* interaction sites per leaf from three different plants (*n* = 3) were evaluated for PEN3‐GFP FA patterns. Those images showing a clear focal accumulation pattern were used for quantification. Fiji was used to draw the FA as a region of interest, which was then analyzed for the mean intensity and the area. Evaluation of callose accumulation was performed as previously described by Underwood (2022). Briefly, three Col‐0 and *ipcs2*_W205_* plants were inoculated with *Bgh*. One leaf per plant was cleared and stained with aniline blue (Lipka et al., 2005). Fifty *Bgh* appressoria per leaf were analyzed for presence or absence of aniline blue‐stained callose.

### Protein work

Total protein extraction from Arabidopsis leaves and Western blot analysis were performed according to a previously published protocol (Petutschnig et al., 2010). The signal intensity and sensitivity of the anti‐GFP (ChromoTek) antibodies were increased using the SuperSignal™ Western Blot Enhancer Kit (Thermo Fisher Scientific) following the manufacturer's instructions.

### Statistical analysis

Statistical differences between different genotypes were calculated via one or two‐way ANOVA (*P* < 0.05) or a two‐tailed *t*‐test (*P* < 0.05).

## ACCESSION NUMBERS

Sequence data for the genes mentioned in this article can be found in the TAIR data libraries under the following accession numbers: IPCS2, AT2G37940; IPCS1, AT3G54020; IPCS3, AT2G29525; EDS1, AT3G48090; PAD4, AT3G52430; SID2, AT1G74710.

## AUTHOR CONTRIBUTIONS

VL, IF, and YB supervised the project; JM, VL, TMH, CH, IF, NE, and YB designed the research; JM, TMH, CH, and NE performed the experiments; JM, TMH, CH, IF, and VL analyzed the data; JM wrote the original manuscript with input from TMH and VL. VL edited the paper with input from all authors.

## CONFLICT OF INTEREST

The authors declare no competing interests.

## Supporting information


**Figure S1.** Mapping of *ipcs2*_W205_*.
**Figure S2.** IPCS2 and not IPCS1 is the major contributor to Arabidopsis cell entry control against non‐adapted powdery mildews.
**Figure S3.** Complementation analyses of *ipcs2*_W205_*.
**Figure S4.** PI4P accumulates at *Bgh* contact sites.
**Figure S5.** Callose deposition at *Bgh* penetration sites is IPCS2‐independent.
**Figure S6.** The spontaneous lesion phenotype of *ipcs2*_W205_* is SA‐dependent.
**Table S1.** Oligonucleotides used in this study.

## Data Availability

The data presented in this study are available in the article or supplementary material. Correspondence and requests for materials should be addressed to vlipka@gwdg.de.
