# Supplementary material for: Identification of INOSITOL PHOSPHORYLCERAMIDE SYNTHASE 2 (IPCS2) as a new rate‐limiting component in Arabidopsis pathogen entry control
Source: Plant J. 2025 Apr 29;122(2):e70159. doi: 10.1111/tpj.70159 (PMC12039476; doi:10.1111/tpj.70159)
Supplement: Supplementary file 1 — Figure S1. Mapping of ipcs2 W205 *. Figure S2. IPCS2 and not IPCS1 is the major contributor to Arabidopsis cell entry control against non‐adapted powdery mildews. Figure S3. Complementation analyses of ipcs2 W205 *. Figure S4. PI4P accumulates at Bgh contact sites. Figure S5. Callose deposition at Bgh penetration sites is IPCS2‐independent. Figure S6. The spontaneous lesion phenotype of ipcs2 W205 * is SA‐dependent. Table S1. Oligonucleotides used in this study. [file TPJ-122-0-s001.docx]

**SUPPORTING INFORMATION**

Supplemental Figure 1: Mapping of *ipcs2_W205*_*.

Supplemental Figure 2: IPCS2 and not IPCS1 is the major contributor to Arabidopsis cell entry control against non-adapted powdery mildews.

Supplemental Figure 3: Complementation analyses of *ipcs2_W205*_*.

Supplemental Figure 4: PI4P accumulates at *Bgh* contact sites.

Supplemental Figure 5: Callose deposition at *Bgh* penetration sites is IPCS2-independent.

Supplemental Figure 6: The spontaneous lesion phenotype of *ipcs2_W205*_* is SA-dependent.

Supplemental Table 1: Oligonucleotides used in this study.


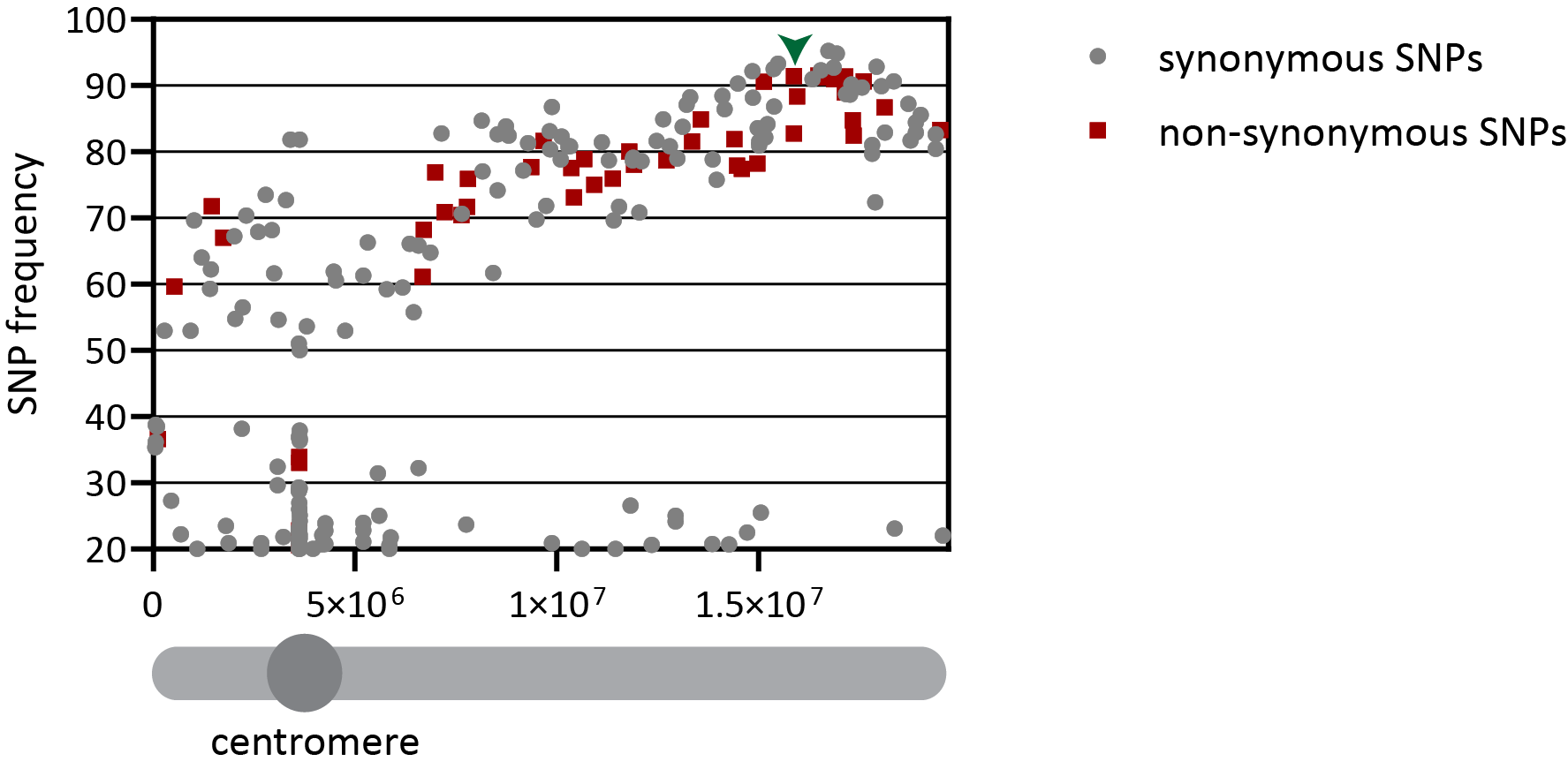


**Supplemental Figure 1: Mapping of *ipcs2_W205*_*.** *ipcs2_W205*_* was backcrossed to Col-0 and the F2 generation was screened again for a *pen* phenotype. DNA of 28 plants with a clear *pen* phenotype was pooled and used for Illumina sequencing. After alignment of the reads to the TAIR10 genome, single nucleotide polymorphisms (SNPs) were called. The frequencies [%] of *ipcs2_W205*_* SNPs were plotted against their position [bp] on the respective chromosomes. This figure shows the frequency peak identified for chromosome 2. Synonymous mutations are shown as grey circles and non‐synonymous mutations as red squares. The causative *ipcs2_W205*_* mutation in *AT2G37940* is indicated by a green arrowhead.

**
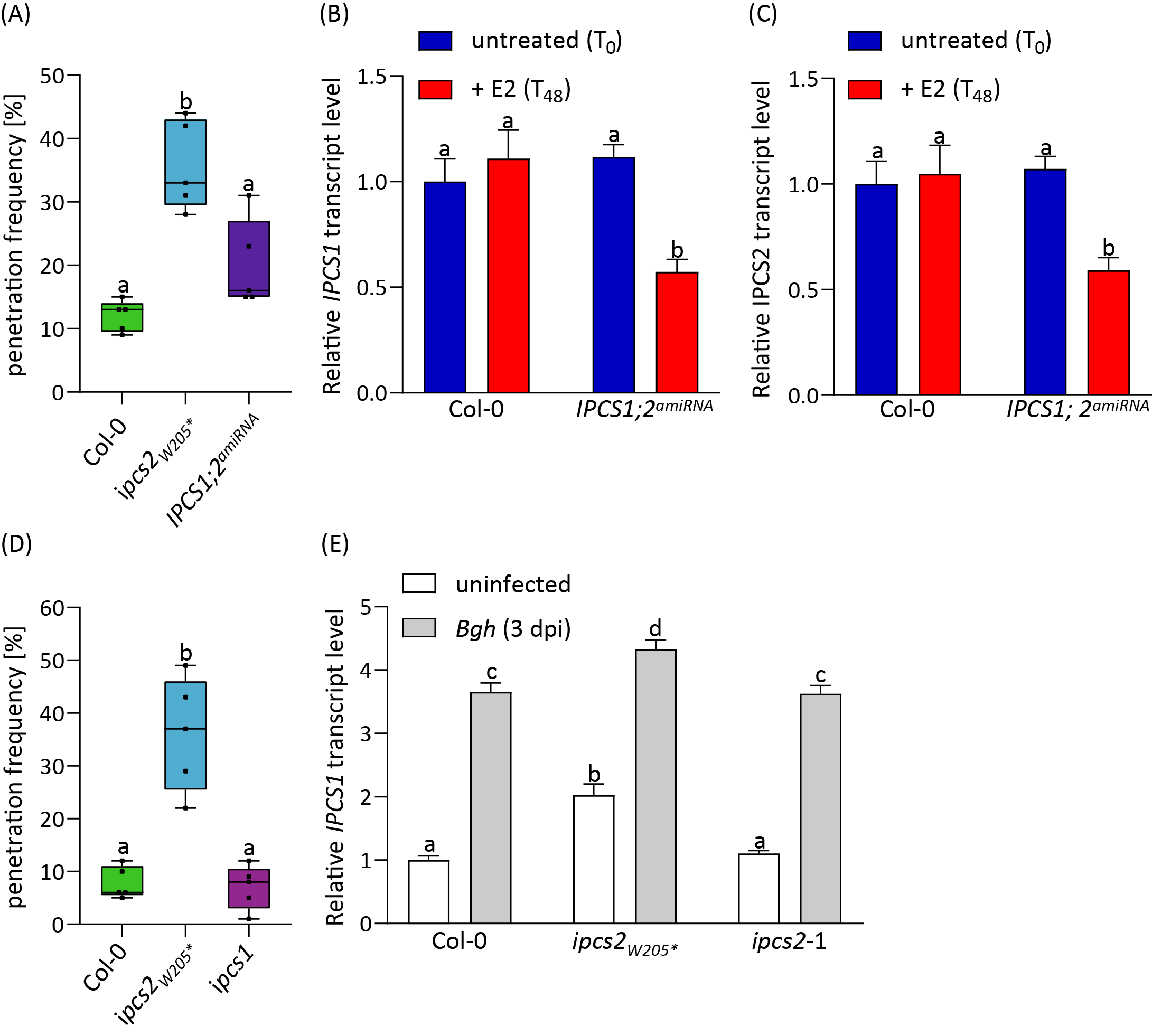
**

**Supplemental Figure 2: IPCS2 and not IPCS1 is the major contributor to Arabidopsis cell entry control against non-adapted powdery mildews.** (A) Col-0, *ipcs2_W205*_* and plants of the *IPCS1;2^amiRNA^* line were sprayed with β-Estradiol (E2) and 48 hours later infected with *Bgh*. Individual boxplots illustrate the penetration frequency of *Bgh* at 3 days post infection (dpi) and whiskers demonstrate minimum and maximum values. Letters show significant differences between genotypes (n =5; two-way ANOVA with Tukey´s post-hoc test; p < 0.05). The experiment was repeated twice with similar outcomes. (B, C) Leaves of wild-type Col-0 and the *IPCS1;2^ariRNA^* line were harvested before (T_0_) and after E2-spraying (T_48_) and were analyzed by quantitative real-time PCR. Presented (B) *IPCS1* or (C) *IPCS2* transcript levels are relative to the untreated Col-0 sample. The bars represent the average ± standard error of the mean (n = 4-5), while letters indicate significant differences between genotypes and treatments (two-way ANOVA with Tukey´s post-hoc test; p < 0.05). Similar results were obtained in an additional independent experiment. (D) *Bgh* penetration frequency on wild-type Col-0, *ipcs2_W205*_* and *ipcs1* mutant leaves at 3 dpi. Individual box plots include whiskers representing minimum and maximum values. Letters show significant differences between genotypes (n = 5; one-way ANOVA with Tukey´s post-hoc test; p<0.05). Similar results were confirmed in an additional independent experiment. (E) Uninfected (white bars) or *Bgh*-infected (3 dpi) (grey bars) leaves of wild-type Col-0, *ipcs2_W205*_* and *ipcs2*-1 were used for quantitative real-time PCR analysis. Presented *IPCS1* transcript levels are relative to the uninfected Col-0 sample. The bars represent the average ± standard error of the mean (n = 4-5). Letters show significant differences between genotypes and treatments (two-way ANOVA with Tukey´s post-hoc test; p < 0.05). The experiment was repeated twice with similar outcomes.


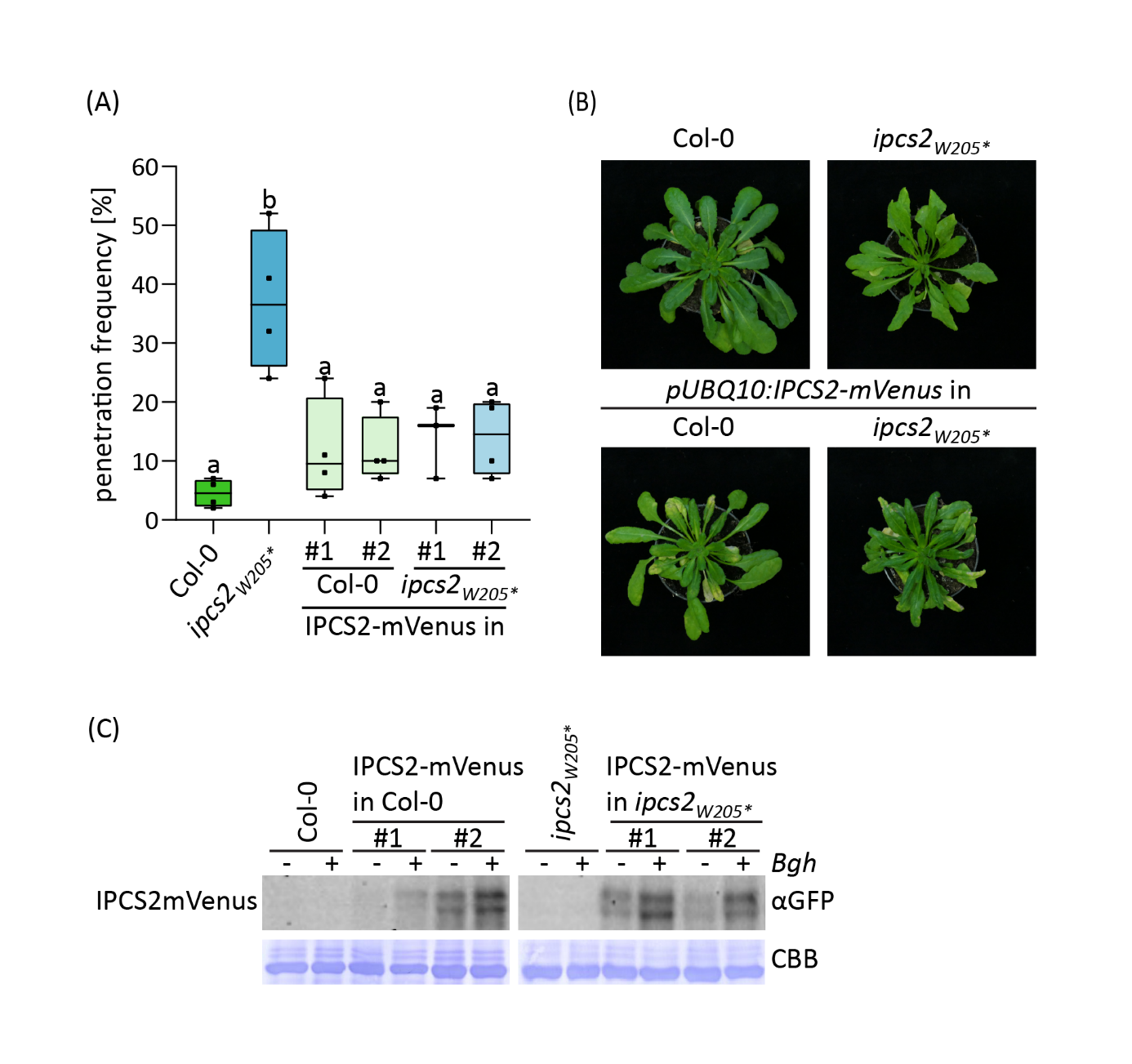


**Supplemental Figure 3: Complementation analyses of *ipcs2_W205*_*.** (A) Col-0, *ipcs2_W205*_* and two independent transgenic plant lines stably expressing *pUBQ10:IPCS2-mVenus* in Col-0 or in the *ipcs2_W205*_* mutant background were infected with *Bgh*. Individual box plots represent the penetration frequency of *Bgh* at 3 days post infection. Whiskers show minimum and maximum values. Letters show significant differences between genotypes (n = 3-4; one-way ANOVA with Tukey´s post-hoc test; p<0.05). (B) Macroscopic phenotype of Col-0, *ipcs2_W205*_* and the above described transgenic lines after 8-weeks under short-day conditions. (C) Col-0, *ipcs2_W205*_* and the two above described independent transgenic lines expressing *pUBQ10:IPCS2-mVenus* in either Col-0 or *ipcs2_W205*_* were infected with *Bgh*. Leaf tissue of uninfected or *Bgh*-infected (24 hours post infection (hpi)) plants were used for total protein extraction and further Western Blot analysis using anti-GFP antibodies. Coomassie brilliant blue (CBB) staining was performed as a loading control. All results shown were replicated in an additional independent experiment.


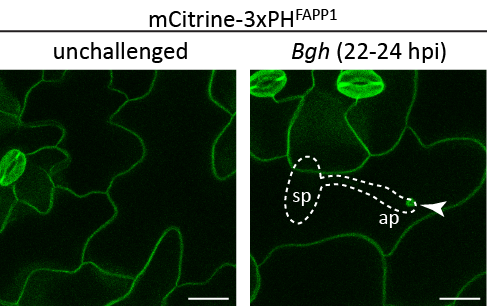


**Supplemental Figure 4: PI4P accumulates at *Bgh* contact sites.** Maximum z-projections of CLSM images from unchallenged (left panel) or *Bgh*-infected (22-24 hours post infiltration (hpi); right panel) leaf epidermal cells of the PI4P biosensor marker line mCitrine-3xPH^FAPP1^. Arrowheads indicate *Bgh* penetration sites, while white lines illustrate fungal spore with appressorium formation. Similar results were obtained in an additional independent experiment. Ap = appressorium; sp = spore. Scale bar = 20 µm.


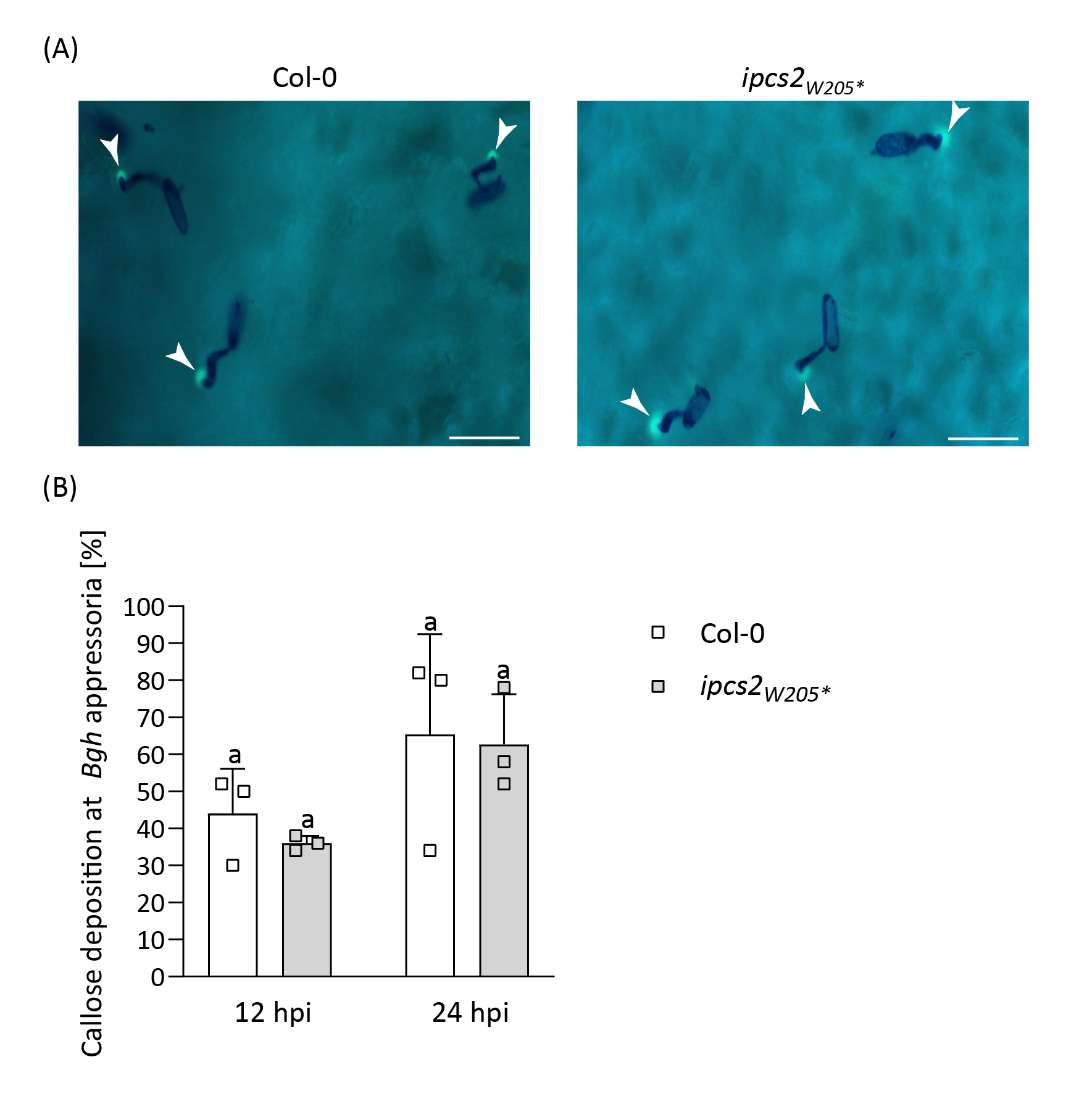


**Supplemental Figure 5: Callose deposition at *Bgh* penetration sites is IPCS2-independent.** (A) Micrographs of aniline-blue- and Coomassie-brilliant-blue-stained leaves of Col-0 (left panel) or *ipcs2_W205*_* (right panel) after infection with *Bgh* (24 hours post infection (hpi)). Arrowheads indicate callose-enriched papillae at *Bgh* penetration sites. Scale bar = 50 µm. (B) Frequency of callose deposition at 50 *Bgh* appressoria at 12 hpi and 24 hpi in Col-0 (white bars) or *ipcs2_W205*_* background (grey bars). Bars represent averages of three biological replicates ± standard deviation. Letters indicate significance at p < 0.05 determined by one-way ANOVA with Tukey's post-hoc test. Experiment was repeated thrice with similar outcomes.


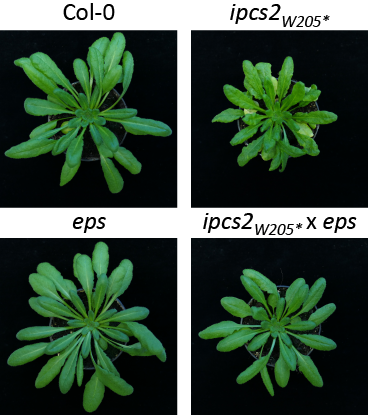


**Supplemental Figure 6: The spontaneous lesion phenotype of *ipcs2_W205*_* is SA-dependent.** Macroscopic phenotype of Col-0, *ipcs2_W205*_*, *eps* and the *ipcs2_W205*_* x *eps* double mutant after 8-weeks under short-day conditions.

**Supplemental Table 1: Oligonucleotides used in this study.**

| **Primer** | **Sequence (5´-3´)** | **Use (Reference)** |
| --- | --- | --- |
| JM157  JM158 | TTTCTAGGTTGCCATGGCCC  GCAACACAGCAGTCCGATCT | Genotyping of *ipcs2_W205*_* by sequencing |
| JM169  JM170 | TAGCCAAATTTGTTTTGCACG  ATCTGAAAAACCCAGGATTCG | Genotyping of *ipcs2*-1 (with EP63) |
| AV70  AV71 | TGCATACTTTTTCATGGAGGG  TTGGGATTTGTTTGCTTTGTC | Genotyping of *ugnt1*-4 (with EP63) |
| EP63 | GCGTGGACCGCTTGCTGCAACT | SALK left border primer |
| JM231  JM280 | TACCAGATCGGACTGCTGTG  CAATCAGCAGGGTCAACACC | qPCR of *IPCS2* |
| EP223  EP224 | GGTTTTCCCCAGTGTTGTTG  CTCCATGTCATCCCAGTTGC | qPCR of *ACTIN8* |
| JM315  JM314 | CTCACTGCAATTGTGCAGAG TCACCCAAACAGCTTTATGAACCTT | Cloning p426GPD-IPCS2_W205*_ |
| JM313  JM314 | GTCGACCTCGAGTCATGTAATTAGT  TCACCCAAACAGCTTTATGAACCTT | Cloning p426GPD-IPCS2_∆205-305_ |
